# Exploring Internal Ribosome Entry Sites as Therapeutic Targets

**DOI:** 10.3389/fonc.2015.00233

**Published:** 2015-10-20

**Authors:** Anton A. Komar, Maria Hatzoglou

**Affiliations:** ^1^Department of Biological, Geological and Environmental Sciences, Center for Gene Regulation in Health and Disease, Cleveland State University, Cleveland, OH, USA; ^2^Department of Pharmacology, School of Medicine, Case Western Reserve University, Cleveland, OH, USA

**Keywords:** eukaryotic translation initiation, IRES, drug, inhibition, disease relevance, disease treatment

## Abstract

Initiation of eukaryotic mRNA translation may proceed via several different routes, each requiring a different subset of factors and relying on different and specific interactions between the mRNA and the ribosome. Two modes predominate: (i) so-called cap-dependent initiation, which requires all canonical initiation factors and is responsible for about 95–97% of all initiation events in eukaryotic cells; and (ii) cap-independent internal initiation, which requires a reduced subset of initiation factors and accounts for up to 5% of the remaining initiation events. Internal initiation relies on the presence of so-called internal ribosome entry site (IRES) elements in the 5′ UTRs of some viral and cellular mRNAs. These elements (often possessing complex secondary and tertiary structures) promote efficient interaction of the mRNA with the 40S ribosome and allow for internal ribosome entry. Internal initiation of translation of specific mRNAs may contribute to development of severe disease and pathological states, such as hepatitis C and cancer. Therefore, this cellular mechanism represents an attractive target for pharmacological modulation. The purpose of this review is to provide insight into current strategies used to target viral and cellular IRESs and discuss the physiological consequences (and potential therapeutic implications) of abrogation/modulation of IRES-mediated translation.

## Introduction

Eukaryotic cells utilize several modes to initiate translation of their messenger RNAs (mRNAs). The most commonly used modes are canonical cap-dependent initiation and internal initiation (1, 2), although several other mechanisms have also been proposed to take place (3, 4). It is generally believed that cap-dependent initiation is responsible for about 95–97% of all translation initiation events in eukaryotic cells and that internal initiation accounts for about 3–5% of the remainder (5–7).

Canonical cap-dependent initiation involves several major steps (1, 2). It starts with activation of the mRNA, which exits the nucleus as an mRNP complex (8), and needs to be mobilized for translation (1, 2, 5–8), and ends with assembly of the elongation-competent 80S complex at the AUG codon. mRNP activation is initiated by binding of eukaryotic initiation factor 4F (eIF4F), which consists of three proteins: (i) eIF4E, the cap-binding protein; (ii) eIF4G, the scaffolding protein that serves as a bridge between the mRNA and the 40S ribosome via interaction with 40S-bound eIF3; and (iii) eIF4A, the ATP-dependent helicase. mRNA activation leads to ATP-dependent removal of secondary structures and proteins from the 5′ end of the mRNA (1, 2). This is followed by recruitment of the 40S ribosomal subunit and associated initiation factors, forming the so-called 43S initiation complex [composed of a 40S subunit, the ternary complex eIF2–GTP–Met-tRNAi^Met^ (TC), eIF3, and eIF1/1A] to be bound to the 5′ m^7^G cap structure of the mRNA (1, 2). The 43S complex then scans the mRNA in search of the initiation codon, where the 48S pre-initiation complex is formed. Following recognition of the start codon and eIF5-induced irreversible hydrolysis of eIF2-bound GTP, eIF5B promotes joining of the 60S ribosomal subunits with the 40S subunits and formation of the elongation-competent 80S complex (1, 2).

Although the vast majority of mRNAs are translated via the mechanism described above, several viral and eukaryotic cellular mRNAs were found to be translated via internal initiation, a process that involves 5′-cap-independent binding of the 40S ribosomal subunits to specific mRNA regions termed internal ribosome entry sites (IRESs) (1, 3–5). Although this translation initiation mechanism is generally independent of recognition of the 5′ mRNA cap structure, it may also involve scanning in search of an initiation codon. Thus, two general modes of IRES-mediated translation have been described/proposed: so-called “direct landing” (whereby the 40S ribosome lands directly onto the AUG codon) and “land and scan” (whereby the 40S ribosome lands in the vicinity of the AUG, but then scans a certain distance to find the AUG) (9).

Poliovirus and encephalomyocarditis virus (EMCV) were the first biological systems found to utilize internal ribsome entry mechanisms to initiate translation of their mRNAs (10, 11). Soon after the discovery of the polio and EMCV IRESs, IRESs were found in families of many other viruses (12, 13). Investigation of viral IRESs showed that IRES-driven translation initiation operates and prevails under conditions when cellular cap-dependent initiation is severely compromised, thus favoring expression of viral mRNAs (1, 5–7, 9). Inhibition of host protein synthesis during viral infection is usually caused by cleavage and partial loss of activity of the eIF4G scaffolding protein (14–16), 4E-BP dephosphorylation resulting in sequestration of eIF4E in the eIF4E-4E-BP complex (17, 18) and/or cleavage of poly(A)-binding protein (PABP) (19, 20), which binds the poly(A) tail of the mRNA and eIF4G and facilitates initiation via circularization of the mRNA (1, 20). Consistent with this, it was found that viral IRESs require a reduced number of translation initiation factors (especially of the eIF4-“family”) with substantial variability depending upon the mRNA, from requiring almost all factors, similar to cap-dependent mRNA translation, to requiring none (1, 13). For example, the hepatitis A virus (HAV) IRES is believed to require almost all canonical initiation factors for efficient translation initiation (21, 22), while the hepatitis C virus (HCV) IRES does not require any of the initiation factors of the eIF4-“family” (1, 23, 24) and the cricket paralysis virus (CrPV) IRES-containing mRNA is translated without the requirement for any of the canonical initiation factors (1, 24–26). It was also found that the majority of viral IRESs possess defined secondary and tertiary structures, which allow for their efficient interaction with the 40S ribosome. This interaction may be direct or partially indirect, requiring the assistance of both some canonical initiation factors and ITAFs (IRES trans-acting factors). ITAFs are known to assist in recruitment of the 40S ribosomal subunit to the mRNA through specific interactions or stabilization of specific active conformations of the IRES (1, 13, 24).

Soon after IRES elements were identified in viral 5′ UTRs (10, 11), it was suggested that cellular mRNAs might also be translated via a cap-independent translation initiation mechanism (27–29) and, indeed, IRESs were identified in a cohort of cellular mRNAs (5, 30). Like viral IRES-containing mRNAs, cellular mRNAs containing IRES elements were found to be preferentially translated under conditions of inhibition of cap-dependent initiation, such as endoplasmic reticulum (ER) stress, hypoxia, nutrient limitation, mitosis, and cellular differentiation. (5, 6). Nevertheless, cellular IRES elements may differ from their viral counterparts in several characteristic features in that they appear to be less structured and not able to bind the 40S ribosomal subunit directly (5, 6). At least, in contrast to viral IRESs, the ability of cellular IRESs to directly bind 40S ribosomal subunits and form a correct initiation complex has not been yet confirmed by initiation complex reconstitution experiments using purified components (4–6). Other characteristic features, such as reduced requirement for canonical initiation factors and/or requirement for specific ITAFs (often shared between viral and cellular IRESs), appear to be quite similar in viruses and eukaryotic cells (5, 6). However, in an attempt to provide a different explanation for a mechanism that could allow some cellular mRNAs to be translated under conditions of inhibition of cap-dependent initiation, the so-called cap- and IRES-independent scanning mechanism of translation initiation was proposed as an alternative to the concept of cellular IRESs (4). Clearly, “cap-independent initiation” does not necessarily mean IRES-dependent initiation in its original (viral-type) sense.

Nevertheless, regardless of the exact molecular mechanism(s) involved, it is clear that initiation of translation of eukaryotic mRNAs may proceed via several different (non-cap-dependent) routes, each requiring a different subset of factors and relying on different specific interactions between the mRNA (harboring specific internal ribosome “landing” regions like IRESs) and the ribosome (1, 5–7, 30). This suggests that translation driven by IRES elements such as viral IRESs that differ in the complexity of their secondary structures or utilize different subsets of initiation factors and ITAFs in comparison with cellular mRNAs might represent attractive targets for pharmacological modulation.

Below, we review current strategies used to target viral and cellular IRESs and discuss the physiological consequences (and potential therapeutic implications) of abrogation/modulation of IRES-mediated expression.

## Strategies to Target IRESs

**Figure 1:**
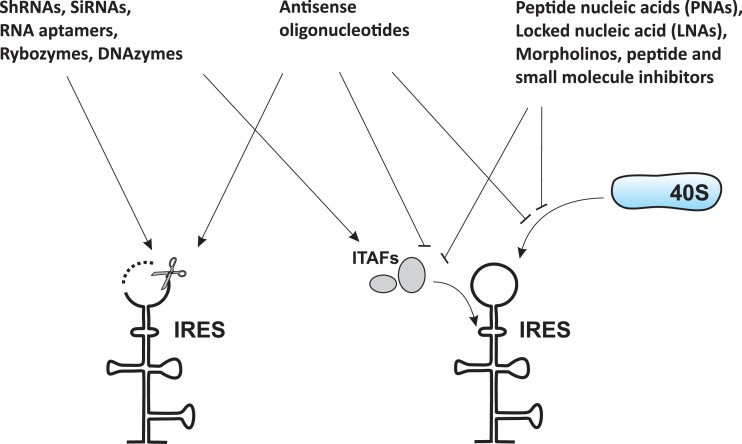
**Common approaches to target IRES-mediated translation**. Several approaches have been developed or are currently under development to target IRES-mediated translation. These approaches include, but are not limited to, use of antisense oligonucleotides, peptide nucleic acids (PNAs), locked nucleic acids (LNAs), morpholinos, short hairpin RNAs (shRNAs), small interfering RNAs (siRNAs), RNA aptamers, ribozymes, DNAzymes, peptides, and small-molecule inhibitors. These agents can cause either destruction of the IRES itself (left) or prevention of IRES interaction with the ribosome and/or protein factors (such as ITAFs) necessary for IRES function (right).

Soon after the discovery of viral IRESs (especially the HCV IRES element), efforts have been made to target them for therapeutic gain (31–33). Due to the limited options generally available to treat viral infections, the design of antagonists able to target specific RNA elements that control the expression of viral proteins, such as IRESs, is of great interest and importance. Such attempts have been primarily focused on the design of antagonists/drugs that will disrupt the IRES itself or prevent IRES interactions with the ribosome or with protein factors (such as canonical initiation factors and ITAFs) necessary for IRES function (Figure 1) (31–41).

Internal ribosome entry site-targeting approaches that have been developed or are currently under development are described in Figure 1 and Table 1. These include, but are not limited to, the use of antisense oligonucleotides [see Ref. (36) for a review], peptide nucleic acids (PNAs) [see Ref. (37) for a review], locked nucleic acids (LNAs) [see Ref. (37) for a review], morpholinos (42, 43), short hairpin RNAs (shRNAs) (41, 44–47), small interfering RNAs (41, 44–47), RNA aptamers, ribozymes (Rz) [see Ref. (48–51) and references therein], DNAzymes (Dz) (52, 53), peptides (54, 55), and small-molecule inhibitors (56–62). While most of these attempts have been focused on preventing/treating viral infections, the overall approach is also believed to represent a potential strategy for cancer prevention and treatment. Rationale for this includes the fact that HCV infection is a major cause of development of hepatocellular carcinoma (63) and many cellular IRES-containing mRNAs (e.g., c-Myc) are known to be implicated in cancer development (64). Therefore, IRES elements are also considered attractive anticancer therapeutic targets (65).

**Table 1:** **Compounds and approaches used to target IRESs**.

Compound	Mechanism of action	Advantages	Disadvantages	Reference
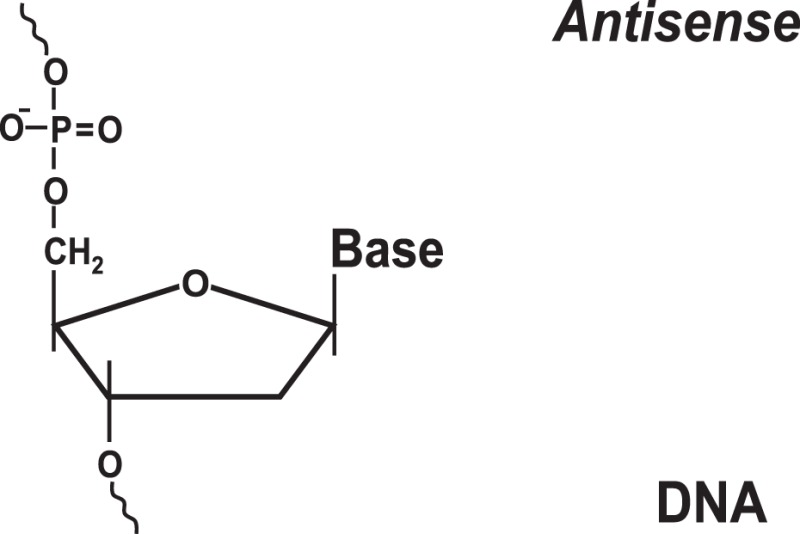	Guide destruction of IRESs/RNAs via an RNAse H-dependent degradation mechanism, or prevent IRESs interaction with the components of the translation machinery (40S ribosomal subunits, ITAFs, etc.)	Easy to design, prepare/obtain	Reduced efficiency of delivery, low intracellular stability, may cause proinflammatory responses	(31, 32, 35, 36, 41, 66)
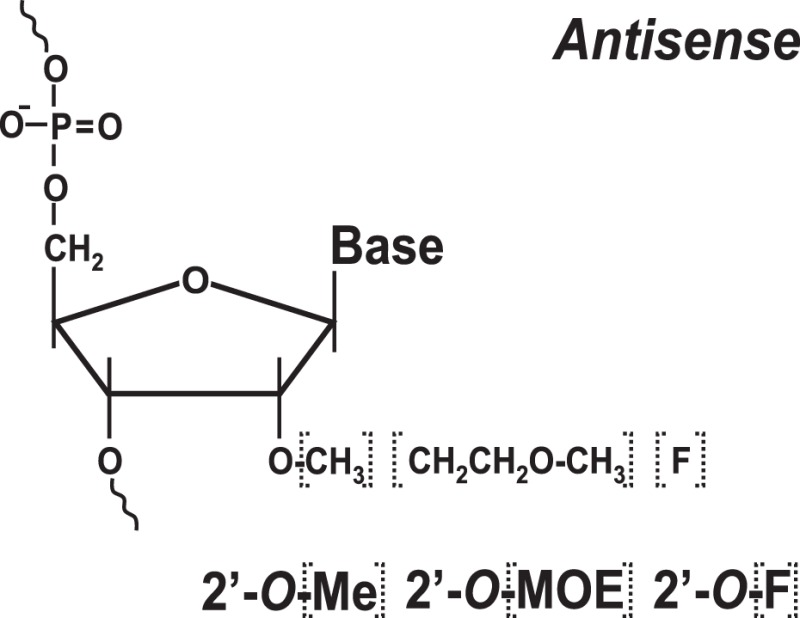	Prevent IRESs interaction with the components of the translation machinery (40S ribosomal subunits, ITAFs, etc.)	Enhanced stability (these compounds are not substrates of RNAse H)	Reduced efficiency of delivery may cause proinflammatory responses	(31, 32, 35, 36, 41, 66)
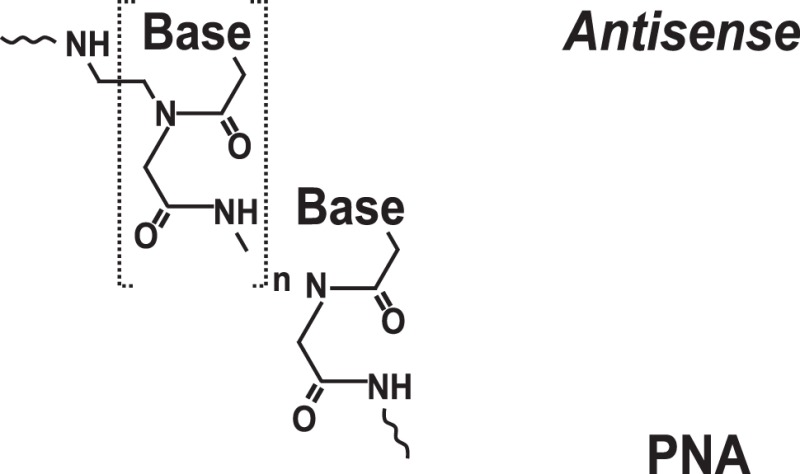	Prevent IRESs interaction with the components of the translation machinery (40S ribosomal subunits, ITAFs, etc.)	Enhanced stability, enhanced affinity toward target RNA sequences	Reduced efficiency of delivery, intracellular trafficking. May be toxic	(36, 37, 67–71)
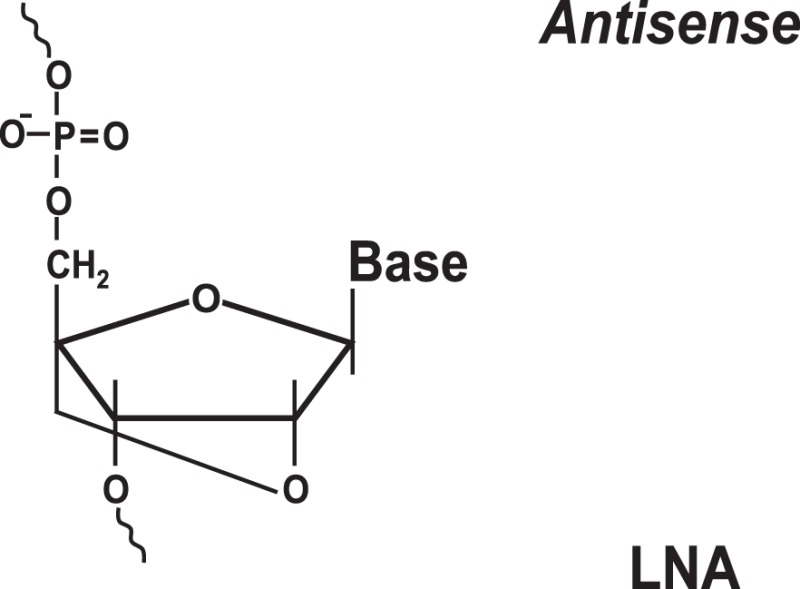	Prevent IRESs interaction with the components of the translation machinery (40S ribosomal subunits, ITAFs, etc.)	Enhanced stability, enhanced affinity toward target RNA sequences	Reduced efficiency of delivery, intracellular trafficking. May be toxic	(36, 37, 67–71)
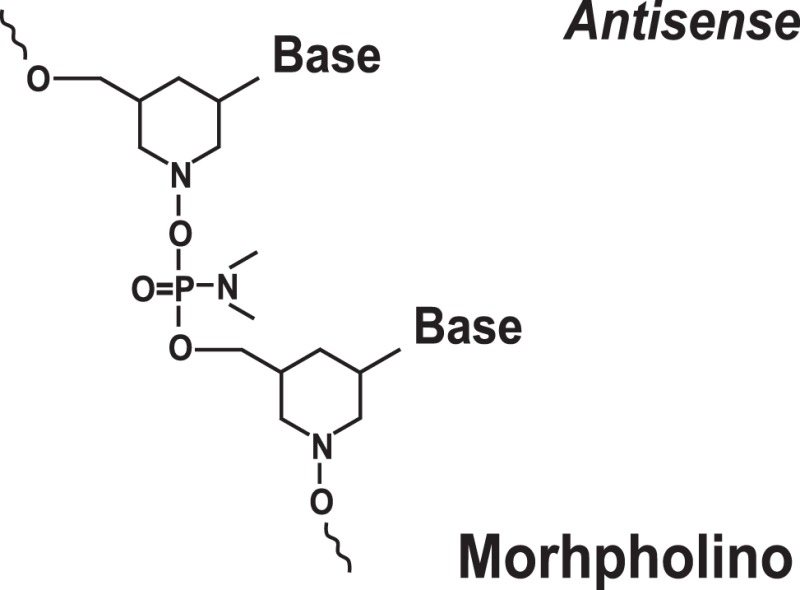	Sterically block target RNAs. Prevent IRESs interaction with the components of the translation machinery (40S ribosomal subunits, ITAFs, etc.)	Enhanced stability, reduced toxicity	Reduced efficiency of delivery, intracellular trafficking. Specificity may be an issue	(42, 43)
Antisense: siRNA, shRNA (RNAi)	Guide destruction of target IRESs/RNAs or mRNAs coding for ITAFs via RISC-dependent mechanism	Easy to design, prepare/obtain	Reduced stability and efficiency of delivery. Specificity may be an issue. May activate PKR	(41, 44–47, 72–75)
RNA aptamers, ribozymes (Rz), DNAzymes (Dz)	Cleave target IRESs/RNAs.	High selectivity	Design process may be complicated	(48–53)
Short peptides, small molecules	Prevent IRESs interaction with the components of the translation machinery (40S ribosomal subunits, ITAFs, etc.)	Considered as the preferred form of drug therapies. Allow lead optimization	Sometimes mechanism of action is difficult to establish and characterize, e.g., when small molecules have been selected during high-throughput screening	(54–62, 76–78)

## Targeting IRES Elements with Antisense Oligonucleotides and Their Derivatives

### Antisense Oligonucleotides

Historically, antisense oligonucleotides were the first agents used to target IRES-mediated translation. These initial attempts were mostly aimed at inhibition of HCV gene expression (31, 32), although EMCV and polio IRESs also served as targets (36). Two types of approaches were used. The first utilized antisense oligonucleotides that guided destruction of viral IRESs/RNAs via an RNAse H-dependent degradation mechanism. This mechanism required the use of phosphodiester-linked (natural) or phosphorothioate-linked nucleotides, which could serve as RNAse H substrates (36). The second approach utilized modified antisense oligonucleotides (2′-*O*-methyl, 2′-*O*-methoxyethyl, 2′-fluoro-, 2-*O*-propyl, etc.) (Table 1) that are not substrates of RNAse H (36). These oligonucleotides were specifically designed to prevent IRES interactions with the ribosome (36). Antisense oligonucleotides of varying lengths (usually 14–28-mers) targeting different structural and functional regions of the IRES (e.g., the HCV IRES) were tested in cell-free *in vitro* system(s) (31, 36), *ex vivo* cellular system(s) (32, 36), and *in vivo* animal models [see Ref. (36, 41) for a review]. The possibility of inhibiting HCV IRES translation by the use of both RNase H-competent and RNase-H incompetent antisense oligonucleotides has been demonstrated [see Ref. (36) for a review]. In the case of the HCV IRES, the most efficient oligonucleotides were found to be those targeting the so-called IIId loop of the IRES, responsible for IRES-40S ribosomal contacts (36), or the region of the mRNA containing the AUG codon (36).

Unfortunately, the approaches described above have several common drawbacks (36) related to the efficiency of delivery of oligonucleotides, their intracellular stability, and in some cases, side effects (such as proinflammatory responses) induced by their use (66).

### Targeting IRES Elements with Peptide Nucleic Acids and Locked Nucleic Acids

To increase the stability as well as the affinity of antisense oligonucleotides, PNAs and LNAs have been developed (36, 37). PNAs are nucleic acid analogs with a neutral 2-aminoethylglycine backbone (79) (Table 1). LNAs contain a methylene group between the 2′-oxygen and 4′-carbon of the ribose ring (79, 80) (Table 1). Thus, LNAs are more conformationally restricted while PNAs remain relatively flexible (79, 80). Both bind complementary sequences with high affinity. PNAs and LNAs are stable to digestion with nucleases/proteases and thus are believed to offer features superior to regular antisense oligonucleotides (79, 80). Results demonstrated that PNAs and LNAs can inhibit IRES-mediated expression *in vitro* and *ex vivo* in cultured cells (36, 37). LNA-based oligonucleotides were also shown to affect viral propagation in HCV-infected chimpanzees (67). However, in the latter case, HCV propagation was affected via an unusual mechanism involving miR-122 molecules that were targeted by LNAs (67). miR-122 binds the HCV 5′ UTR and forms a complex that promotes viral RNA stability and replication (68, 69). Blocking interaction between miR-122 and the HCV mRNA resulted in marked suppression of HCV RNA propagation (67). Several companies are currently developing LNA-based anti-miR-122-based therapeutics for advanced clinical trials (70). Despite these encouraging results, delivery and intracellular trafficking of such modified oligonucleotides remains a limitation of this methodology (36, 37). In addition, some (but not all) studies reported toxic effects associated with the use of LNAs (71).

### Targeting IRES Elements with Morpholinos

Morpholinos are third-generation modified antisense oligonucleotides that have favorable toxicity profiles and also possess increased nuclease stability (79). Morpholinos carry bases that are bound to morpholine (diethylenimide oxide 1,4-oxazinane tetrahydro-1,4-oxazine) rings instead of deoxyribose rings and that are linked together via phosphorodiamidate groups (79) (Table 1). Morpholino–RNA duplexes are more stable than their corresponding DNA–RNA duplexes. Morpholinos act by steric blocking of the target RNA sequences and are widely used to modulate gene expression in several model organisms, such as zebrafish and frogs (79). Morpholino antisense oligonucleotides (usually 20–25-mers) were found to be potent inhibitors of HCV IRES-mediated translation *in vitro* and in a preclinical mouse model (42). These morpholinos were designed to target the HCV IRES region near the AUG codon (42). Inhibition was specific for the HCV IRES and not the EMCV IRES (42). A set of peptide-conjugated phosphorodiamidate morpholino oligomers (PPMO) were also developed against conserved IRES sequence found in picornoviruses, such as human rhinovirus type 14, coxsackievirus type B2, and poliovirus type 1 (PV1) (43). These PPMOs were found to efficiently inhibit virus replication in cultured cells (43). Moreover, treatment of poliovirus type 1-infected mice resulted in reduced PV1 titers in tissues of the central nervous system and protection from a lethal outcome (43).

Difficulty achieving efficient delivery to the target cells and intracellular trafficking remains a major obstacle precluding wide use of morpholinos as well as the other oligonucleotide-based approaches discussed above. Moreover, a recent report suggests that morpholino off-target effects may be much more prevalent than previously thought (81). This reiterates the importance of careful validation of any oligonucleotide-induced phenotype (81).

The antisense-based technologies described above typically target loop regions of IRES elements in order to maximize the affinity and binding efficiency between the antisense oligonucleotide(s) and the RNA (36–38). Other regions amenable to targeting include unpaired joint sequences, hairpins, and bulges (36–38). Surprisingly, only moderate variation in targeting efficiency (less than twofold) between loops, hairpins, and unpaired joint sequences has been reported (37).

### Targeting IRES Elements with Short Hairpin RNAs and Small Interfering RNAs

In recent years, RNA interference (82) has been widely used to inhibit gene expression. High conservation of the HCV IRES and similar viral IRES elements make them attractive targets for RNA interference (41, 44–47). Both small interfering RNAs (siRNAs) and shRNAs have been successfully used to suppress HCV IRES expression in cultured cells and model animals (e.g., mice with humanized liver) (41, 44–47). A similar approach was also used to target cellular IRES elements or cellular ITAFs (hnRNP A1, HuR, etc.) required for the function of IRESs (including cellular IRESs) (72, 73). However, a key challenge associated with RNAi-based therapeutics is similar to those associated with oligonucleotide-based approaches: difficulty in achieving efficient delivery of the material to the target cells. In addition to stability issues, efficient entry of the RNAi molecules into cells is hampered by their negative charges. To overcome this obstacle, various lipid-based systems including, but not limited to liposome- and polymer-based nanoparticles, have been developed [see Ref. (74) and references therein].

The question of whether RNA interference has significant advantages over antisense-based technologies is not easy to answer. It seems that at present, oligonucleotide-based approaches look more advantageous due to higher intracellular stability of DNA-based oligonucleotides and, in many cases, their higher affinity toward selected targeted RNA regions. One of the additional drawbacks of RNAi-based technologies is that siRNAs and shRNAs may activate protein kinase R (PKR) (75), thus leading to inhibition of host cell translation due to phosphorylation of translation initiation factor eIF2-alpha (1, 75).

### Targeting IRES Elements with RNA Aptamers, Ribozymes, and DNAzymes

Ribozymes, DNAzymes, and RNA aptamers (linked to hammerhead ribozymes) have also been used to target IRES sequences, again with the HCV IRES being the main focus (48–53). DNAzymes are catalytic DNA molecules that can be designed to cleave target RNAs (similar to ribozymes) in a sequence-specific manner [see Ref. (83) and references therein]. It is believed, however, that synthetic DNAzymes are easier to prepare than synthetic ribozymes (83) and, in addition, DNAzymes are more stable (83). Unlike siRNAs and shRNAs, DNAzymes are not expected to activate PKR (52). Various ribozymes, RNA aptamers fused with ribozymes, and DNAzymes have been tested for their ability to reduce expression of IRES-driven reporter constructs (mostly HCV IRES-based) and to inhibit viral replication in cultured cells and mouse models (48–53). Several sites in IRESs are usually targeted in order to maximize cleavage by catalytic nucleic acids (48–53). Promising results showing the selectivity and specificity of inhibition of IRES-mediated expression by ribozymes and DNAzymes suggest that they may serve as potent therapeutic agents against viral IRES-driven translation (48–53).

### Peptide and Small-Molecule Inhibitors

Short peptides and small molecules are widely used as drugs (84–86). Short peptides are composed of 5–40 amino acids and usually mimic selected biological activities of the full-length proteins from which they were derived (84, 85). Some drugs in clinical use are short peptides (e.g., Fuzeon, which is an inhibitor of HIV-1 cell entry) (87).

Several peptides have been developed that target the HCV IRES (41, 54, 55). These peptides were mostly derived from the La-autoantigen (specifically, from its RNA recognition motif RRM) and were shown to abrogate La-HCV IRES binding (54) (La is an ITAF of the HCV IRES) (88). The La-derived peptides were shown to moderately inhibit IRES-driven translation *in vitro* and in cultured cells (54, 55). However, one issue with this strategy is that since La serves as an ITAF for many viral and cellular IRESs (5, 6, 88, 89), the effect of these peptides would likely not be specific for the HCV IRES.

Small molecules are currently considered the preferred form for drug therapies (86). Novel approaches to synthesize collections of compounds (libraries) have revolutionized our ability to generate large numbers of related small molecules rapidly and on demand (86). It has recently been found that a number of nucleic acid intercalating agents are capable of inhibiting IRES-mediated initiation of translation to a much greater extent than cap-dependent initiation (56). However, screening attempts for IRES-binding inhibitors have been hampered by the complex and sometimes dynamic architecture of these elements, which makes them especially difficult to target using small molecules. On the other hand, it is precisely this complex and distinct architecture that makes IRES elements attractive for targeting. Numerous attempts have been made by a number of companies and research centers to address this problem [see Ref. (56–62) and references therein]. As a result, a number of potent small-molecule inhibitors able to specifically suppress some viral [e.g., HCV, EMCV, and polio (56, 58, 59, 61, 62)] and cellular IRESs (e.g., c-Myc and VEGF) have been identified (76). The so-called benzimidazole inhibitors (targeting the HCV IRES basal domain IIa) were shown to suppress viral replication in cell cultures at micromolar concentrations with low toxicity (61). Aminoglycoside-based compounds were also found to be effective (62). Several such inhibitors have progressed to clinical trials. For example, VGX-410C (from VGX Pharmaceuticals) appeared to be safe in phase 2 trials, but was later found not to be effective (77).

Despite such setbacks, the approach is still considered to have strong potential and, as mentioned above, was also applied to modulate cellular (e.g., c-Myc and VEGF) IRES-driven translation (76). Small molecule hits shown to modulate c-Myc-IRES expression in a reporter construct *in vitro* were also tested *ex vivo* and shown to decrease c-Myc protein expression and modulate the viability of ovarian cancer cells (76). The exact mechanism of action of many of these small-molecule drugs on IRES-driven translation is, however, not well understood (76). It is believed that many of these drugs act by intercalating into IRESs and preventing binding of mRNAs to the ribosome (78). However, the extent of inhibition varies substantially with different intercalating drugs, showing dependence on the structural complexity of both the IRES and the drug (56, 58, 78). Unfortunately, at present, there is limited understanding of why certain intercalating drugs preferentially inhibit IRES-mediated translation compared to others. Nevertheless, the accumulated data suggest that mRNA structural complexity is likely a critical determinant of this process (56, 58, 78).

### Physiological Consequences and Importance of Targeting IRES-Mediated Translation

While the physiological consequences and benefits of targeting viral IRES-mediated translation are quite obvious and should generally lead to abrogation of viral infections (34–36, 38, 40, 41), the outcome of downregulation (or upregulation) of cellular IRES-mediated expression is less clear. Downregulation of cellular IRES-mediated expression may lead to severe disease states like X-linked dyskeratosis congenita (X-DC) (90), a condition characterized by bone marrow failure, skin abnormalities, and increased susceptibility to cancer (91). Therefore, selection of appropriate targets and understanding of the exact effects of regulation of cellular IRES-mediated expression is extremely important. Nevertheless, cellular IRES-mediated expression represents an attractive therapeutic target, particularly for diseases (such as some cancers) that are resistant to conventional therapies (65). Below, we provide a brief overview of the physiological states and consequences associated with regulation of cellular IRES-mediated translation.

**Figure 2:**
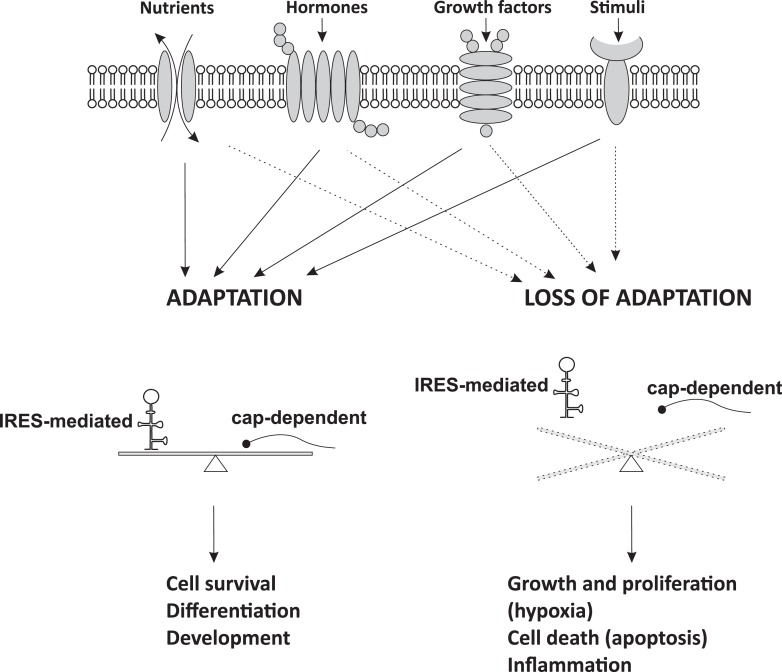
**IRES-mediated translation and cell fate**. Cells respond to extracellular signals and changes in intracellular homeostasis by regulation of mRNA translation. Adaptive IRES-mediated translation is usually balanced with cap-dependent mRNA translation and is essential for cellular function. When adaptation starts failing, cap-dependent mRNA translation usually decreases and IRES-mediated translation prevails. This switch in translational control is the beginning of disease development leading to cellular dysfunction. Dysregulation of cap-dependent translation in cancer states may result in both up- and down-regulation of cap-dependent translation and imbalanced IRES-mediated expression.

Regulation of IRES-mediated translation of cellular mRNAs depends on the intracellular and extracellular environment (5–7). As discussed earlier, a general rule is that signaling pathways that inactivate cap-dependent translation are expected to promote IRES-mediated translation (5–7). IRES activity-promoting signaling pathways are usually associated with cellular stress conditions, such as hypoxia, inflammation, tumorigenesis, and growth factor responses (5–7). Therefore, cap-dependent and IRES-mediated translation should be carefully balanced in cells (Figure 2).

An example of how signaling is important for physiological stress and reprogramming of cellular translation is hypoxia associated with increased expression of HIF-1α protein, which is known to reprogram metabolism from oxidative to glycolytic modes (92), thus limiting reactive oxygen species (ROS) production through oxidative phosphorylation (92). In this way, ROS levels and oxidative damage of proteins are limited in tissues such as the brain. Interestingly, HIF-1α is also associated with a switch from cap-dependent to adaptive IRES-mediated translation (93). The interesting finding that deletion of PTEN-induced putative kinase-1 (PINK1) promotes this translational switch, via mechanisms that involve HIF-1α, suggests that therapeutics can be used to promote IRES-mediated translation in tissues like the brain for the treatment of neurodegenerative diseases such as Parkinson’s disease (94). Generation of ROS in the early onset of Parkinson’s disease is believed to contribute to its rapid progression (94).

Another example in support of the importance of the physiological regulation of IRES activity is the finding that homeobox (Hox) mRNAs contain IRES elements in their 5′ UTRs and these mRNAs have developed a self-sufficient mechanism to suppress cap-dependent translation of their own mRNAs (95). This mechanism involves the presence of an RNA regulon in their 5′-UTRs that inhibits their cap-dependent translation, thus facilitating organismal development (95). This mechanism of IRES-mediated translation is likely present in other cellular conditions, still awaiting discovery.

Considering that adaptive IRES-mediated translation is protective (discussed above) and disease-induced IRES-mediated translation is undesirable, therapeutics can be developed to target specific IRES elements or signaling pathways that enhance the activity of these IRES elements. A good example of the latter case is the translational reprogramming that occurs in cancer cells that have adapted to hypoxic, nutritionally poor and inflammatory conditions. Several growth- and survival-promoting proteins (89) have been identified as containing IRES elements in the 5′ UTRs of their mRNAs (96). The IRES-trans-acting factors for some of these IRES elements are partially known [recently reviewed in Ref. (96)] and, in some cases, the signaling leading to their activation has been also described (96). A recent report that provides support for the development of therapeutics to inhibit IRES-mediated translation involves the regulation of the IRES activity of the c-Myc mRNA (97). C-Myc is a protein that promotes growth and survival in many cancers. It was shown in multiple myeloma (MM) cells that the activity of the c-Myc IRES was dependent on the MAP kinase MNK1 (MAPK-interacting serine/threonine kinase 1) and the IRES-trans-acting factor HnRNPA1 (an RNA binding protein) (97). This protein is known to be phosphorylated by MNK1 and to act as an IRES-transacting factor for a few mRNAs (98, 99). Interestingly, a small molecule that inhibits interaction of HnRNPA1 with the c-Myc IRES abolished c-Myc IRES-mediated translation (stimulated by stress) and c-Myc protein accumulation (97). These findings are encouraging for the development of small-molecule inhibitors of the interaction between IRESs and their IRES-trans-acting factors as a strategy to inhibit tumor growth.

Another interesting recent report has identified an IRES element in the cyp24a1 (1,25-dihydroxyvitamin D3 24-hydroxylase) mRNA, which encodes a protein that inactivates vitamin D3-mediated signaling (100). This IRES was induced by vitamin D3 and inflammation and involved the PI3K-AKT1 signaling pathway (100). Cyp24a1 was also found to be induced in breast and colon cancer cells and its enhanced expression may therefore explain the development of tumor resistance to chemotherapies in which D3 is used as an adjuvant agent (100). Understanding how inflammation causes translational reprogramming in different cancers will be an important step toward targeting IRES-mediated translation for treatment of these diseases.

Finally, we wish to return to our earlier discussion regarding how interest in targeting IRES-mediated translation first arose. As mentioned above, many IRES elements were found to have reduced requirements for canonical initiation factors, including eIF2 (5–7). In many instances, viral and cellular IRES-mediated translation predominates because eIF2 is inactivated by phosphorylation of its α subunit (eIF2α) by stress-induced eIF2α kinases (99). This inactivation increases the inhibitory interaction of eIF2 with the nucleotide exchange factor eIF2B, thus causing global inhibition of cap-dependent protein synthesis (1, 99). In contrast, under these conditions, IRES-mediated translation prevails. Interestingly, recent reports have shown that therapeutic drugs for diseases that involve cellular responses to stress, including phosphorylation of eIF2, can reverse the inhibitory effects of eIF2 phosphorylation, restore eIF2B guanine nucleotide exchange factor activity, and restore protein synthesis even in the presence of the factors that cause cellular stress (101–104). Given that stress-induced signaling modulates IRES activity of cellular mRNAs, loss of the inhibitory effect of eIF2α phosphorylation may have differential effects on the translation of mRNAs that contain IRES elements. This finding presents a new round of questions regarding the response of cellular IRES-mediated translation to stress under these novel conditions of drug-induced adaptation.

Future studies will bring light to these interesting developments and improve our overall understanding of the precise mechanisms involved in IRES-mediated translation. Nevertheless, it is clear that targeting of this mode of translational regulation holds strong promise as a therapeutic strategy.

## Conflict of Interest Statement

The authors declare that the research was conducted in the absence of any commercial or financial relationships that could be construed as a potential conflict of interest.
